# Adaptation of Microelectrode Array Technology for the Study of Anesthesia-induced Neurotoxicity in the Intact Piglet Brain

**DOI:** 10.3791/57391

**Published:** 2018-05-12

**Authors:** Emily D. Geyer, Prithvi A. Shetty, Christopher J. Suozzi, David Z. Allen, Pamela P. Benavidez, Joseph Liu, Charles N. Hollis, Greg A. Gerhardt, Jorge E. Quintero, Jason J. Burmeister, Emmett E. Whitaker

**Affiliations:** ^1^Department of Anesthesiology, Ohio State University College of Medicine; ^2^Medical Student Research Program, Ohio State University College of Medicine; ^3^Department of Anesthesiology and Pain Medicine, Nationwide Children's Hospital; ^4^Department of Neuroscience, University of Kentucky Medical Center

**Keywords:** Neuroscience, Issue 135, Glutamate, hippocampus, neurotransmitters, neuroinflammation, neurodevelopment, sevoflurane, pediatric anesthesia

## Abstract

Every year, millions of children undergo anesthesia for a multitude of procedures. However, studies in both animals and humans have called into question the safety of anesthesia in children, implicating anesthetics as potentially toxic to the brain in development. To date, no studies have successfully elucidated the mechanism(s) by which anesthesia may be neurotoxic. Animal studies allow investigation of such mechanisms, and neonatal piglets represent an excellent model to study these effects due to their striking developmental similarities to the human brain.

This protocol adapts the use of enzyme-based microelectrode array (MEA) technology as a novel way to study the mechanism(s) of anesthesia-induced neurotoxicity (AIN). MEAs enable real-time monitoring of *in vivo* neurotransmitter activity and offer exceptional temporal and spatial resolution. It is hypothesized that anesthetic neurotoxicity is caused in part by glutamate dysregulation and MEAs offer a method to measure glutamate. The novel implementation of MEA technology in a piglet model presents a unique opportunity for the study of AIN.

**Figure:**
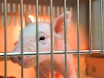


## Introduction

Every year, millions of children undergo anesthesia for both invasive and non-invasive procedures in the United States^1^. For years, anesthesia providers have reassured parents of the safety of anesthetics, even in small children and neonates. However, in 1999 it was found that transient blockage of the N-methyl-D-aspartate (NMDA) subtype of glutamate receptors during early life could cause widespread neuronal apoptosis in rats^2^. Recently, the FDA released a drug safety communication that will require the labels of anesthetic drugs to include a warning about general anesthetics and their potential negative effect on the developing brains of children younger than 3 years old (U.S. Food and Drug Administration, 2017). However, there is still a need to elucidate possible mechanisms and potential neuroprotective measures.

Normal activity of neurotransmitters such as glutamate and gamma-amino butyric acid (GABA) are critical for normal neurodevelopment to occur. Although most of the pathways involved in AIN are still elusive, neurotransmitter systems are very likely to be involved since anesthetics are known to modulate these pathways to produce unconsciousness. In particular, the excitatory neurotransmitter glutamate causes excitotoxicity when its activity is dysregulated. This neurotransmitter is normally involved in neurogenesis, neural plasticity, synaptic and neural growth, and a number of other critically important brain functions. However, prolonged activation of glutamate receptors can cause excitotoxicity and neuronal apoptosis, particularly under stress conditions such as surgery, oxygen deprivation, and reduced energy availability^3^. Binding of glutamate to the NMDA receptor has been shown to cause Na^+^ and Cl^−^ influx. The subsequent depolarization is thought to lead to Ca^2+^ channel opening and release of intracellular Ca^2+ ^stores^4^. This dysfunction likely leads to a cascade of metabolic derangements which eventually decrease neuronal proliferation, increase inflammation, and lead to neuronal death. Despite these hypotheses, the true mechanism(s) of AIN remain unclear^5^. Because of its role in apoptosis, glutamate dysregulation represents a novel pathway that may contribute to the mechanism of previously documented neuronal apoptosis, a feature of AIN.

One of the hindrances on the study of neuronal processes is their high complexity, especially in the setting of neuronal development. The first few months of life are the period of maximal vulnerability to injury, during which most of the important steps of neuronal development such as physiologic apoptosis (neuronal pruning), synaptogenesis, gliogenesis, and myelination take place^6^. Given the complex nature of neuronal communication and the difficulty of studying these processes without disrupting normal CNS function, new technologies have been developed which aim at the *in vivo *detection and quantification of important elements of neuronal communication.

Enzyme-linked MEA technology was used in this study as a novel way to study the mechanisms of AIN in a clinically-relevant piglet model. This technology can be used to study complex* in vivo* electrochemical brain processes, including glutamate dysregulation. The incorporated 4-channel platinum recording sites of the MEAs (2 glutamate-sensitive sites and 2 sentinel sites) allow self-referencing, which contributes to detection accuracy. In addition, an exclusion layer is applied to each electrode, conferring selectivity by preventing other interfering molecules from being detected^7^. Moreover, the low-profile design of the MEA allows for minimal tissue trauma compared to earlier technologies. This same feature confers to MEAs a higher spatial resolution, which facilitates the study of microscopic regions in the brain. For example, discrete regions of the hippocampus (dentate gyrus, CA1, CA2) can be specifically studied^8^. Specific details on the functionality of MEAs have been previously described^9^.

In comparison to MEA electrochemistry, microdialysis incorporates a membrane placed between the solution of interest and a solution of similar composition, allowing detection of extracellular fluid changes^10^. Although microdialysis is a mainstay of neurochemistry and has long been used for the detection of neurotransmitters, it has the disadvantage of low time resolution, delayed detection of glutamate change, and significant tissue trauma^11^.

MEAs can indirectly detect neurotransmitters such as glutamate, acetylcholine, and choline, by using appropriate oxidase enzymes that produce electroactive reporter molecules such as H_2_O_2 _or O_2_^12^,^13^.

MEA technology has been widely used in rats and non-human primates for the study of neurotoxicity in the context of pathophysiological processes other than AIN^7^,^14^. Among some of these pathophysiological processes, MEA technology has been used for the study of Alzheimer's disease, epilepsy, traumatic brain injury, and the effect of pharmacological compounds on synaptic communication^8^,^15^,^16^,^17^. Although MEAs have been used to study these pathologies in rats and non-human primates, the high developmental similarity between humans and piglet brains makes the adaptation of the MEA technology in piglets a highly suitable technique for the study of AIN mechanism(s)^18^.

## Protocol

Piglets (*Sus scrofa*) are received through a local farm pre-approved by The Ohio State University (OSU) Institutional Animal Care and Use Committee (IACUC). Following approval of the protocol, animal experimentations are done in accordance with IACUC policy.

### 1. Piglets and Piglet Handling

1. Utilize male and female piglets in a systematic and randomized manner to eliminate any potential sex-based confounders in accordance with ARRIVE guidelines^19^. NOTE: Since the period of maximal brain growth is within 3-5 days of piglet birth, experimentation is done solely with piglets 3-5 days old.
2. Ensure piglets arrive in the vivarium at least 24 h before experimentation to allow acclimation to the environment. NOTE: Trained veterinary staff provides routine animal care. The piglets are kept in individually temperature-maintained, continuously monitored cages and receive a nutritionally-complete milk replacer *ad libitum *to prevent hypoglycemia. The piglets are also kept without milk replacement (*nil per os*), for at least 3 h prior to anesthesia and are supplied with blankets and toys to ensure normal levels of stimulation. If possible, keep multiple piglets in the same cage to allow socialization.

### 2. Development and Customization of MEAs for AIN Studies in a Piglet Model

NOTE: This technology uses enzyme-based MEAs that are pre-coated with enzyme and electroplated with m-phenylenediamine dihydrochloride (mPD). The electrodes were custom designed with a 40-mm rigid shaft and manufactured for use in piglets (Figure 1).

1. To avoid redundancy, please see the detailed description of MEA preparation and calibration as previously described^15^ (Figure 2).

### 3. Anesthesia and Use of Custom Stereotaxic Apparatus for the Piglet

1. Anesthetize animals using an anesthesia workstation with an appropriate ventilator and monitoring devices, and monitor physiologic parameters such as pulse oximetry, non-invasive blood pressure, electrocardiography, and temperature throughout the entirety of the experiment as previously described^19^. Intubate and ventilate the piglets and administer sevoflurane anesthesia at 1 minimum alveolar concentration (MAC) (approximately 2.5-3%) for 3.5 h. Ensure that trained laboratory staff members are present for these experiments. Fur overlying the surgical area is removed using electric clippers prior to preparation of the skin. NOTE: The concentration and duration of anesthetic used allows the experiment to simulate the time-length of actual anesthesia exposure during a surgical procedure. Additionally, sevoflurane is the most commonly utilized general anesthetic in the pediatric population making the investigation surrounding its safety of utmost importance.
2. Before placement into the stereotaxic frame, start a rocuronium loading dose of 2.5 mg/kg and an infusion of 1.5 mg/kg/h to prevent animal movement while secured in the frame.
  1. Place the piglet in the piglet-specific stereotaxic frame once an adequate depth of anesthesia is confirmed by toe-pinch.
  2. Provide adequate padding within the stereotaxic frame by placing the piglet on a forced- air warming pad with additional padding (*e.g.*, fluidized positioner) to prevent pressure ulcers.
  3. Place the teeth of the upper mandible over the tooth bar (Figure 3). NOTE: The tooth bar should be at a sufficient level to hold the skull very firmly in place.
  4. Fix and tighten the penetrating ear bars within the ears, taking care to ensure that the piglet is in the midline position. Position the pointed tips of the lateral holds within the ear canal and insert the ear bars firmly enough to hear the "pop" sound associated with the penetration of the tympanic membrane. Firmly attach the ear bars to the skull and insert to equal depth on each side in order to prevent movement of the skull during the experiment (Figure 4**, Panel A**). NOTE: It is vitally important to keep the piglet warm (~ 37.8-38.6 °C) and continuously monitor the temperature during the entire procedure for maintenance of normothermia. This can be accomplished *via* a blanket and/or a heat lamp. Be sure to place the heat lamp at an appropriate distance to avoid burning of the animal's skin.
3. Create a 4-6 cm midline incision along the skull using caution to avoid scoring the skull with the scalpel. Once the incision is made, use gentle retraction and blunt dissection to elevate the scalp from the skull. Gently scrub the skull with a gauze pad to remove any connective tissue and expose the suture lines (Figure 4**, Panel B**). NOTE: It is not necessary to maintain sterility during non-survival experiments. However, sterility must be maintained during survival experiments.
4. Further reflect the scalp, if necessary, to expose the area of interest and determine the intended location for the craniotomy (Figure 5**, Panel A**). Use a surgical drill to create a craniotomy window of approximately 0.25 cm^2^ (may be larger or smaller depending on experimental goals) overlying the structure of interest, using caution not to injure the dura or the underlying brain (Figure 5**, Panel B**). Use fine surgical scissors to excise the dura overlying the brain tissue (Figure 5**, Panel C**).
5. Position the electrode as vertically as possible over the bregma by securing the metal arm of the headstage to the micromanipulator of the piglet stereotax. Lower the electrode as much as possible without touching the surface of the skull. Record the coordinates of the bregma (Figure 6**, Panel A**).
  1. Determine the anterior-posterior and medial-lateral coordinates as well as the depth of the structure of interest as they relate to the bregma. Determine the stereotaxic coordinates using a species and age-appropriate stereotaxic atlas. In this case, use a stereotaxic atlas developed specifically for piglets^20^.
  2. Reposition the electrode so that it has the proper anterior-posterior and medial-lateral location, ensuring that both the microelectrode and the apparatus are perpendicular to the surface. Place the pseudo-reference electrode under the scalp, ensuring contact with the animal (Figure 6**, Panel B**).
  3. Slowly and gently, lower the electrode to the appropriate depth into the brain using the stereotaxic arm. For the final 2 mm, use a hydraulic microdrive to further drive the electrode to the exact location (Figure 6**, Panel C**). NOTE: The electrode position should overlie the craniotomy window. The electrode depth will vary depending on the brain structure of interest. It is not necessary to close the incision upon completion of data collection in non-survival experiments.

### 4. Measurement of Extracellular Glutamate Under Sevoflurane Anesthesia

1. Ensure the piglet is under continuous physiologic monitoring throughout the entirety of this procedure. Piglets are anesthetized inhalationally (via face cone) in preparation for the procedure. 
2. After implantation of the MEA, wait 30 min to allow the electrode to reach baseline to ensure that correct measurement will be obtained for 3 h (Figure 7).
3. 0.25% bupivacaine (1 mL/kg) is administered subcutaneously at the site of the surgery for postoperative pain management. In addition, sustained-release buprenorphine is given subcutaneously q72h as needed.

### 5. Perfusion and Sacrifice

1. Perform the perfusion and brain tissue collection procedures as previously described^21^. For non-survival surgeries, animals are euthanized immediately after experimentation while still under general anesthesia.
2. Take gross cross-sections of the fixed piglet brain and use light microscopy to visualize the track of the electrode as previously described^22^ to allow verification of MEA placement, ensuring proper placement in the area of interest.

## Representative Results

This proof-of-concept study with enzyme-based ceramic MEA technology in a piglet model can provide exceptional insight into the glutamate dynamics underlying AIN. This study further demonstrates that enzyme-based MEA technology can be successfully adapted in the piglet model to measure physiologic and anesthesia-associated changes in neurotransmitter activity with high sensitivity, and high spatial and temporal resolution. Physiologic homeostasis was maintained throughout our experiments using clinically relevant methods and standards, and no piglet exhibited signs of physiologic perturbations.

The data obtained indicates the ability of MEAs to precisely and spatially resolve neurotransmitter measurements in cortical and sub-cortical brain structures. The use of a stereotaxic apparatus enables clear identification of a reference surface structure (bregma) in order to consistently locate the region of interest, regardless of individual differences in piglet size and anatomy. Clear visualization of the sutures facilitates consistent regional placement of the MEA with accuracy in the micrometer range (Figure 4). Obtaining access to the cortical surface of the brain is minimally traumatic with negligible bleeding, ensuring that any *in vivo* glutamate dynamics are not due to unintentional systemic or local insult (Figure 5). The custom, rigid MEA is then aligned perpendicular to the frontal plane of the piglet (Figure 6). Failure to properly align the MEA prior to insertion may prevent accurate spatial recording of the targeted region, especially for subcortical regions.

Real-time *in vivo* glutamate measurements were taken in the hippocampi of 3-4-day old piglets (*n *= 4) under 2.5-3% sevoflurane anesthesia (approximately 1 MAC). Amperometry measurements were recorded at 4 Hz and converted to concentration using a linear regression based on calibration parameters (Figure 8**, Panel A**). For each time point, the signals from the two glutamate-sensitive sites were averaged before subtracting the averaged sentinel signal to yield a corrected glutamate signal. These continuous measurements were smoothed by applying a moving average to better visualize the overall trend over time (Figure 8**, Panel B**). The mean basal glutamate concentration was calculated to be 4.61 ± 0.02 µM and remained relatively stable over the course of anesthetic exposure. Transient glutamatergic activity was identified in one animal by analyzing peaks in the signal that were not correlated with the sentinel signal (R^2^ < 0.5) and exceeded a signal-to-noise ratio of 3 (Figure 9**, Panel A**). A total of 116 transient peaks were detected over a period of 3.5 h (Figure 10**, Panel A**). The amplitude of the resulting transient peaks was generally observed to be within the 1 µM range (Figure 10**, Panel B**). In order to quantify the duration of each transient, the time (t_80_) required for each maximum peak value to decay 80% was obtained (Figure 9**, Panel B**). The mean t_80_ of all glutamate transients during the 3.5 h recording period was 4.68 ± 0.82 s (Figure 10**, Panel C**). These data demonstrate that it is possible to accurately measure both prolonged and transient neurotransmitter activity in a subcortical region of the anesthetized piglet brain.

**Figure:**
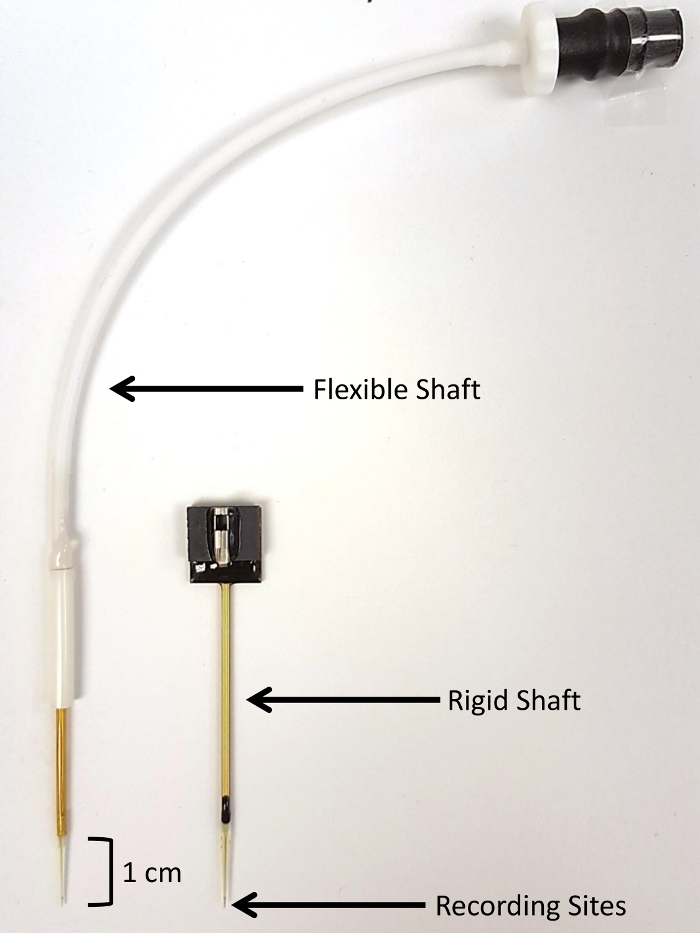

**Figure 1: Visual comparison of SG-2 microelectrode array types.** SG-2 arrays contain two glutamate-sensitive sites and two glutamate-insensitive sentinel sites (150 µm x 20 µm per site).**(A**) A flexible-shaft microelectrode array is shown on the left. The rigid-shaft microelectrode array was custom-designed for use in piglets and permits deeper implantation in large animals. Please click here to view a larger version of this figure.

**Figure:**
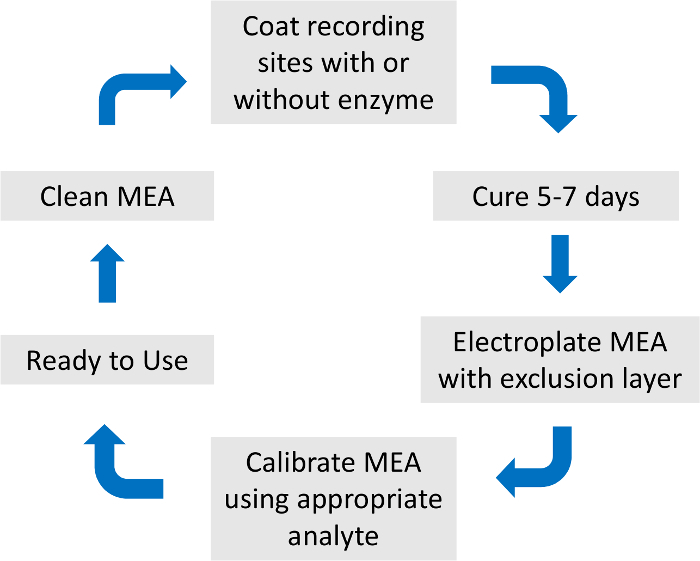

**Figure 2: Overview of microelectrode array preparation and calibration process.** The total MEA preparation and calibration last approximately one week. The coating enzyme, exclusion layer, and calibration analytes are specific to the neurotransmitter of interest. Please click here to view a larger version of this figure.

**Figure:**
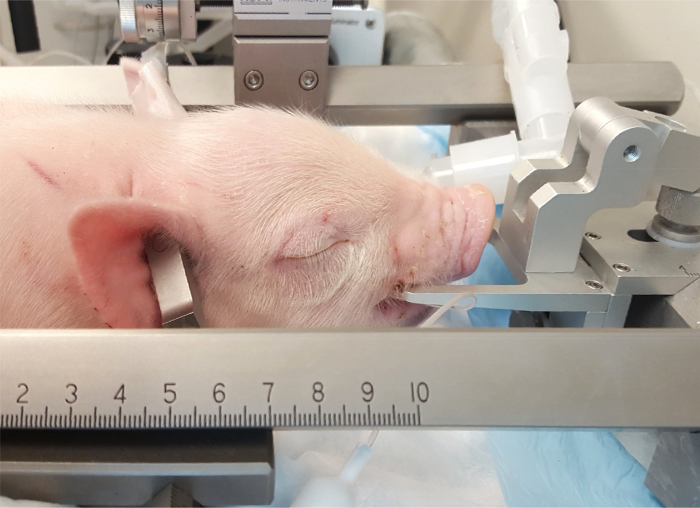

**Figure 3: Placement of piglet in the stereotaxic apparatus.** The piglet mouth is placed on mouth bar directly posterior to the canine teeth. The penetrating ear bars are inserted into the ear canals to secure the posterior end of the skull. Please click here to view a larger version of this figure.

**Figure:**
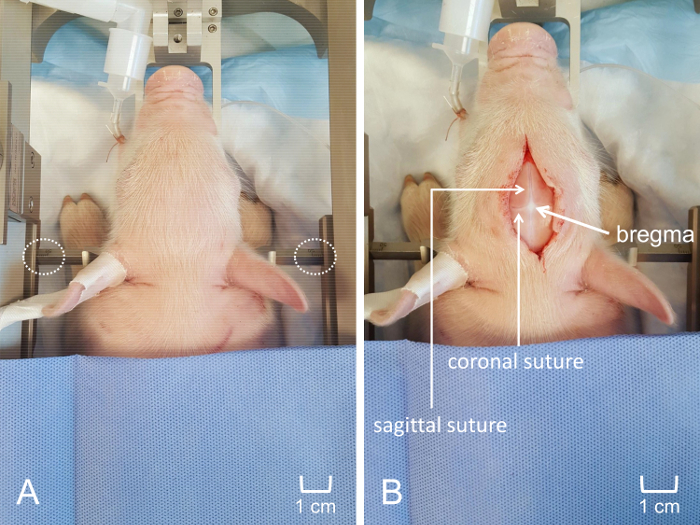

**Figure 4: Placement of piglet in the stereotaxic apparatus for craniotomy.** (**A**) The piglet's head is tightly secured within the custom stereotaxic frame, ensuring consistent placement of the MEA. Equidistant placement of penetrating ear bars is visible. (**B**) Midline anterior-posterior incision along the scalp. Scoring of the skull was avoided to visualize coronal and sagittal sutures and optimize visualization of bregma. The scale bar is shown to indicate the relative size of the incision and the location of craniotomy window. Please click here to view a larger version of this figure.

**Figure:**
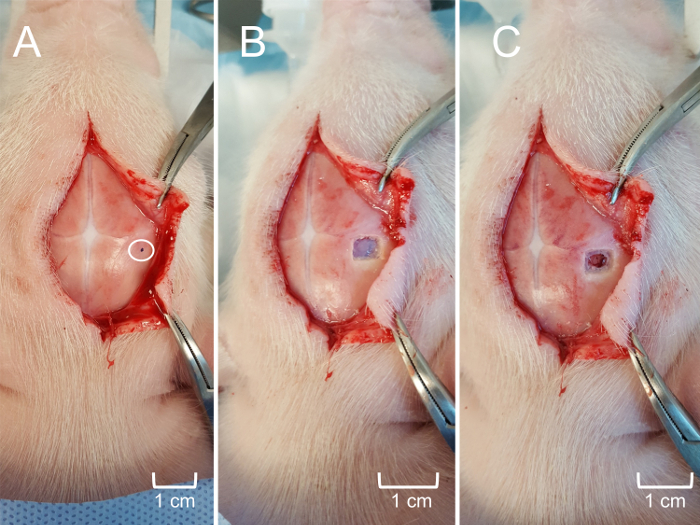

**Figure 5: Craniotomy for access to the hippocampus.** (**A**) The scalp further reflected to expose the approximate location of the MEA insertion according to stereotaxic coordinates. The circled area is marked (black dot) to guide the craniotomy. (**B**) The craniotomy window (0.25 cm^2^) with skull flap removed to expose the underlying dura mater. (**C**) The meninges carefully removed to expose superficial cerebral cortex without tissue trauma. Please click here to view a larger version of this figure.

**Figure:**
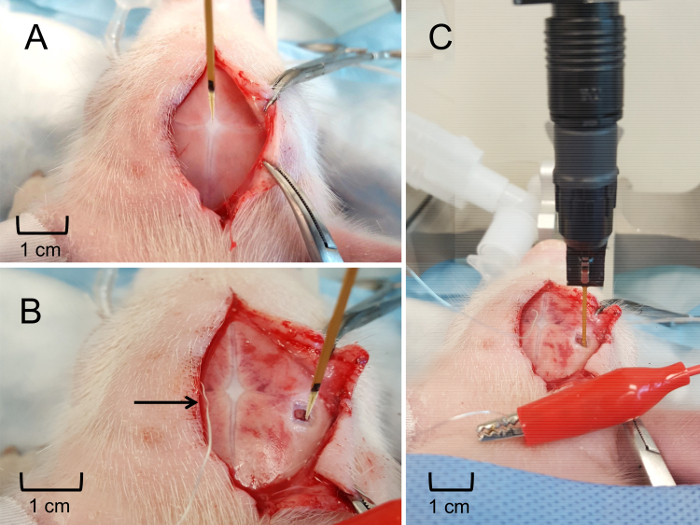

**Figure 6: MEA positioning and insertion into the hippocampus.** (**A**) Placement of MEA at the bregma to determine a relative stereotaxic location of the hippocampus. (**B**) Stereotaxic placement of the MEA at the brain surface to determine the hippocampus insertion depth. Silver pseudo-reference electrode securely placed under scalp (indicated by arrow). (**C**) the MEA inserted at the appropriate depth to obtain real-time, *in vivo* extracellular glutamate measurements in the hippocampus. Please click here to view a larger version of this figure.

**Figure:**
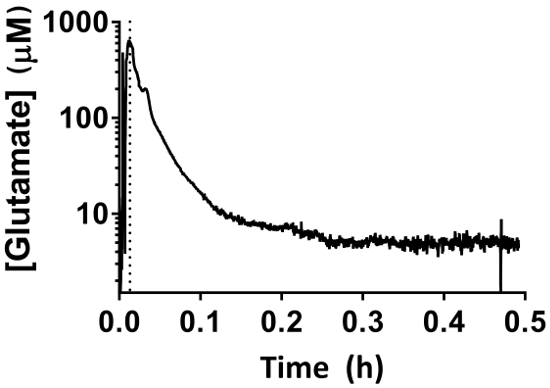

**Figure 7: MEA behavior during the 30-min baselining period.** The initial rapid increase corresponds to the descent of the MEA into the hippocampus using the micromanipulator. The baseline period begins once the MEA has reached the appropriate depth (dotted line). Extracellular glutamate measurements will decrease over a period of 30 min and should not be interpreted as physiologic readings. Please click here to view a larger version of this figure.

**Figure:**
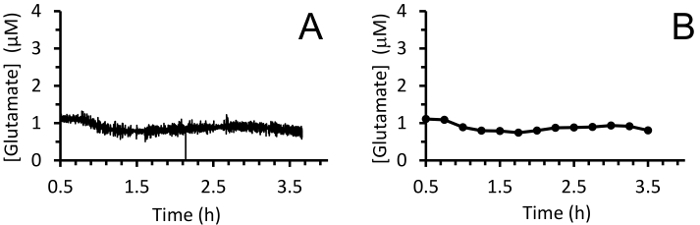

**Figure 8: Real-time extracellular glutamate measurements in the hippocampus of a neonatal pig under sevoflurane anesthesia.** (**A**) The moving average of the glutamate concentration in the hippocampus of one neonatal pig under sevoflurane anesthesia (using 10 data points). Measurements were taken at 4Hz for 3 h after a brief 30 min baselining period. (**B**) Smoothing of glutamate measurements using a moving average of 100 points every 15 min to better visualize the trend. Please click here to view a larger version of this figure.

**Figure:**
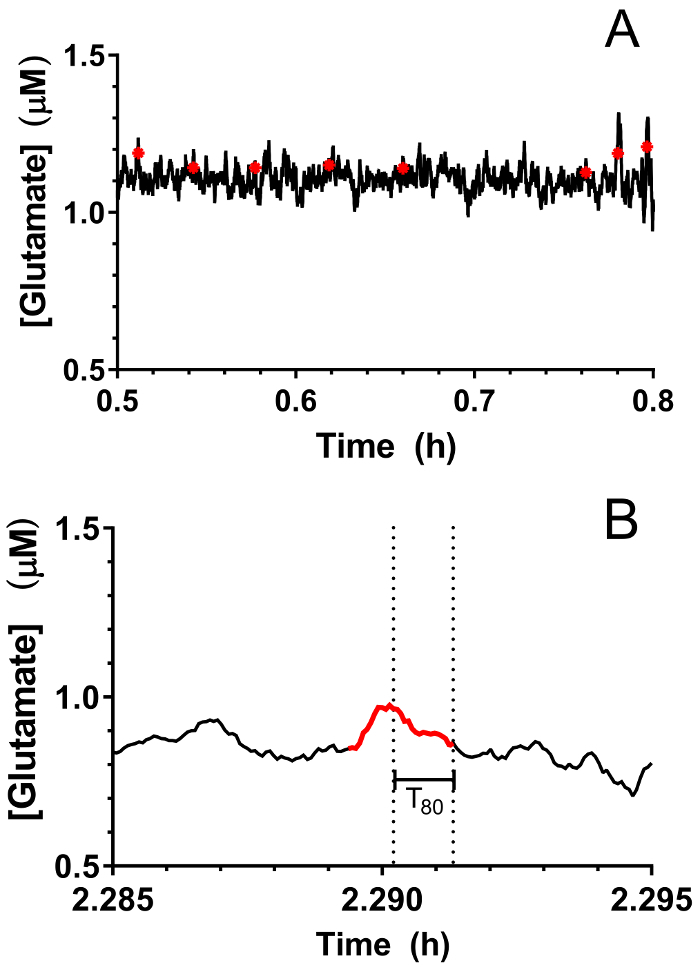

**Figure 9: Identification of transient glutamate activity in the hippocampus of a neonatal pig under sevoflurane anesthesia.** (**A**) Transient glutamate peaks (in red) are indicated on the real-time glutamate tracing. Peaks were considered significant when the signal to noise ratio exceeded 3 and their signal was not correlated with the sentinel signal (R^2^ < 0.5). (**B**) A representative transient peak identified in Figure 9**, Panel A**. Dotted lines indicate the total duration required for the peak to decay 80%. Please click here to view a larger version of this figure.

**Figure:**
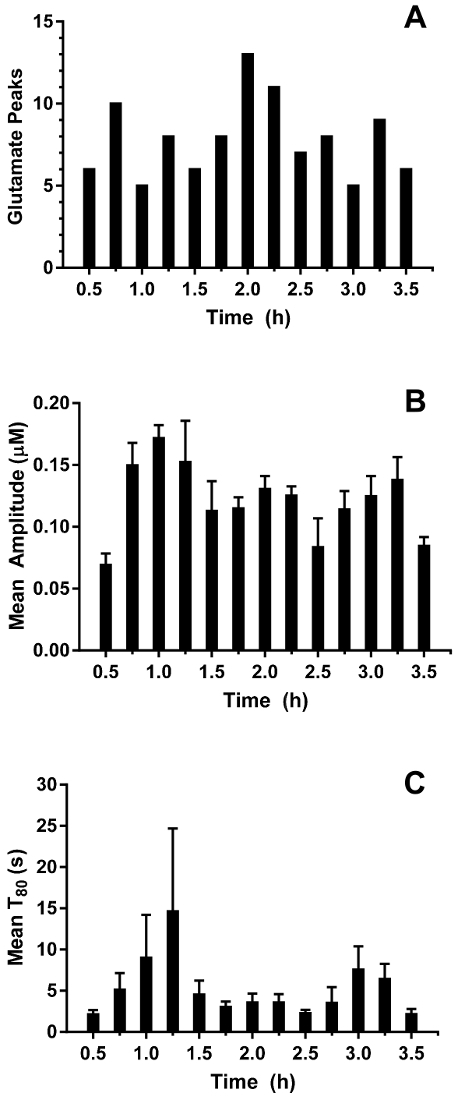

**Figure 10: Characterization of glutamate transients in the hippocampus of a neonatal pig under sevoflurane anesthesia.** (**A**) A number of transient glutamate peaks in 15 min bins. Peaks were considered significant when the signal to noise ratio exceeded 3 and their signal was not correlated with the sentinel signal (R^2^ < 0.5). (**B**) The amplitude of transient glutamate peaks. Error bars indicate the standard error of the mean. (**C**) Mean T_80 _of transient glutamate peaks. Error bars indicate the standard error of the mean. Please click here to view a larger version of this figure.

## Discussion

From the start of the experiment, the piglet's physiological homeostasis must be maintained as described in this lab's prior publication^21^. Minimal monitoring should include pulse oximetry, electrocardiography, capnography, non-invasive blood pressure, and temperature. Trained investigators are required so that physiologic perturbations (*e.g.*, hypo/hyperthermia, hypoxia, hypotension, arrhythmia) can be appropriately corrected.

Prior to induction, *in vitro* MEA calibrations are performed to establish functionality and selectivity of the MEA under known conditions. The calibration and plating of MEAs is critical for effective use of the technology. There are many potential errors that can arise during calibration. Calibration can identify these issues as well as improper plating, which leads to incorrect interferent response. A more detailed tabular account of errors that may occur in MEA response has been compiled, along with notable causes and suggested solutions, which should prove a useful instrument for likely troubleshooting (**Table 1**). It is important to note that prior to both calibration and plating, the glass reference electrode should be checked for the presence of air bubbles or white discoloration, as either will negatively impact MEA function and recording accuracy.

**Table:** 

**Symptom**	**Cause**	**Corrective Action**
No Signal	Electrode not connected	Properly connect electrode to headstage and headstage to FAST amperometry system.
No power to the FAST amperometry system	Turn on power switch on back of FAST system
Signal Noise	Electrode contaminated by blood	Continuously irrigate the brain surface during electrode insertion
Rinse the electrode immediately in dH_2_O
Enzyme coating is loose	Clean and recoat the electrode
Reference electrode was not inserted or coated	Coat and place the reference electrode farther under the scalp
Electrode is detecting movement of brain surface	Usually occurs in superficial structures. Insert the electrode deeper (1 mm at a time) if possible
Animal Movement	Animal is inadequately secured	Move the animal in the posterior direction to better secure earbars on the skull. If necessary, elevate the torso to allow for better body alignment.
Animal is inadequately anesthetized	Verify the integrity of the anesthetic equipment. Titrate the anesthetic to an effective dose and administer an intramuscular rocuronium dose (5 mg/kg)
Inaccurate electrode placement	Electrode is not correctly aligned	Readjust the electrode while maintaining proper connection to the headstage.
Stereotaxic coordinates are inaccurate	Ensure that the piglet atlas being referenced does not use another reference point or plane of alignment.
Be careful not to obscure the suture marks by scoring the skull.

**Table 1: Instructions for troubleshooting MEA use in piglets. **Possible causes and corrective actions to assist with optimization and troubleshooting.

A stereotaxic atlas for the piglet is used to determine the stereotaxic coordinates of the area of interest with respect to a known point such as bregma^18^. Ear bars should be properly secured to ensure that the skull is level and fully immobilized. Care should be taken during the midline incision of the scalp to avoid scoring the skull as this may affect visualization of the suture lines. The craniotomy window should be large enough to accommodate the MEA.

This protocol presents a number of technical challenges that require a well-stocked operating suite and an investigator/team skilled in the surgical and anesthetic aspects of the protocol. The model additionally presents financial limitations in that the piglet model is more expensive than the rodent model; however, it is significantly less costly than the use of non-human primates, which can cost thousands of dollars. The use of MEA technology presents its own challenges, as the procedure of coating and plating the electrodes manually require a skilled investigator or assistant to ensure sufficient selectivity and reliable function. The microelectrodes themselves are fragile, as they are made of ceramic, and thus easily damaged if proper caution is not observed. Microelectrodes are subject to interference from other electrical devices, which can create noise in recordings, and from blood at the operative site, which can occlude the recording sites. The need for specialized equipment presents an additional burden as a stereotaxic surgical frame must be custom built to immobilize the piglet skull during implantation. The stereotaxic frame, glutamate oxidase, and the electrodes themselves are all costly. Additionally, the lack of a piglet stereotaxic atlas from within the last decade poses technical limitations that require particular expertise to determine the specific location of deep structures in the piglet brain. Development of a new stereotaxic atlas, perhaps using magnetic resonance imaging, would greatly enhance the ability to use this technology in piglets.

The piglet is a clinically relevant model for the study of AIN largely due to the parallels that exist between this species and the human neonate, as both possess similar brain structure and development. Unlike more commonly used models such as mice or rats, the piglet has a greater CNS similarity to humans, which lends to the translatability of the model's results. The piglet model is additionally cheaper and involves less complicated handling than a non-human primate model. The piglet model is intended to examine the process by which anesthesia might induce developmental neurotoxicity, measure its contribution to neurological damage, and combat the issue of damage caused by confounding variables. For instance, hypoxia may be misconstrued for damage caused by anesthetics as it has global effects on the brain. The piglet is utilized with the same surgical and anesthetic conditions as those used in human medicine to ensure fidelity of results.

The use of ceramic-based MEA technology eliminates several of the disadvantages associated with the contemporary technique of microdialysis. Microdialysis has limited temporal and spatial resolution in comparison to amperometric methods such as the MEA, which can continuously record glutamate events in multiple, microscopic regions at up to 10 Hz^23^. This rapid sampling rate eliminates the confounding factor of localized neurotransmitter diffusion that is inherent to slow-sampling methods like microdialysis^24^. Additionally, the MEA is a less invasive method than a microdialysis probe, which can cause significant gliosis during insertion and may alter neurotransmitter activity at the insertion site^22^.

Previous studies utilizing a range of mammalian models, measurement techniques, and regions of the brain, have demonstrated basal glutamate levels comparable to those found using this technique. This suggests that MEA technology, when adapted to the piglet model, provides valid recordings of *in vivo *glutamate concentration (**Table 2**).

**Table:** 

**Author (Year)**	**Recording Technique**	**Animal Model**	**Age**	**Brain Region(s)**	**Mean Basal Glutamate Concentration (µM)**
Hascup et al. (2008)^23^	MEA (Enzyme-based)	Rodent	20 - 24 weeks	Prefrontal Cortex, Striatum	3.3 ± 1.0; 5.0 ± 1.2
Hascup et al. (2010)^25^	MEA (Enzyme-based)	Rodent	3 - 6 months	Hippocampus	4.7 - 10.4
Rutherford et al. (2007)^9^	MEA (Enzyme-based)	Rodent	3 - 6 months	Prefrontal Cortex, Striatum	44.9 ± 4.7; 7.3 ± 0.9
Miele et al. (1996)^26^	Microdialysis (Enzyme-based)	Rodent	-	Striatum	3.6 ± 0.5
Day et al. (2006)^27^	MEA (Enzyme-based)	Rodent	3 - 6 months	Frontal Cortex, Striatum	1.6 ± 0.3 ;1.4 ± 0.2
Quintero et al. (2007)^28^	MEA (Enzyme-based)	Non-Human Primate	5.3 - 5.5 years	Premotor Cortex, Motor Cortex	3.8 ± 1.7; 3.7 ± 0.9
Stephens et al. (2010)^29^	MEA [Spencer-Gerhardt-2 (SG-2)]	Non-Human Primate	11 - 21 years	Putamen	8.53
Kodama et al. (2002)^30^	Microdialysis (Enzyme-based)	Non-Human Primate	-	Prefrontal Cortex	1.29 - 2.21
Galvan et al. (2003)^31^	Microdialysis (Enzyme-based)	Non-Human Primate	Juvenile	Striatum	28.74 ± 2.73
During and Spencer (1993)^32^	Microdialysis (Enzyme-based)	Human	18 - 35 years	Hippocampus	20.3 ± 6.6
Reinstrup et al. (2000)^33^	Microdialysis (Enzyme-based)	Human	-	Frontal Cortex	16 ± 16
Cavus et al. (2005)^34^	Microdialysis (Enzyme-based)	Human	15 - 52 years	Neocortex	2.6 ± 0.3

**Table 2. Comparison of basal extracellular glutamate levelsacross various animal models. **A selected review of studies establishing normal extracellular glutamate levels in healthy awake and anesthetized animals using microdialysis or microelectrodes.

The use of MEA technology to monitor *in vivo* glutamate concentrations in the piglet model can allow for the future evaluation of piglet neurological outcomes post-anesthesia. Survival experiments have been planned, which will further an understanding of the long-term impact of anesthesia on the neurocognitive well-being of human neonates. Survival experiments will allow for behavioral testing and monitoring of glutamate changes long after anesthesia exposure. It is also common for children to undergo anesthesia in conditions where they might experience physiological stress in the form of surgical intervention. Future studies addressing the influence of surgery in terms of neurological injury and increase in neurotoxicity would allow for more accurate modeling of a common clinical setting for children. The use of alternate animal models is also feasible, as is the study of these various models through chronic implantation, allowing us to track behavioral changes associated with neurotoxicity. MEA technology itself is versatile, so future study need not be limited to analysis of glutamate levels (*e.g.*, GABA, choline, lysine, *etc. *could be analyzed).

## Disclosures

Greg Gerhardt is the principal owner of Quanteon LLC. Jorge Quintero and Jason Burmeister have served as consultants to Quanteon LLC.
